# Supplementary material for: Evaluation of the delivery of the family nurse partnership programme in Scotland during the COVID-19 pandemic
Source: PLoS One. 2025 Dec 2;20(12):e0337080. doi: 10.1371/journal.pone.0337080 (PMC12671793; doi:10.1371/journal.pone.0337080)
Supplement: Attachment — Submitted filename: Response to reviewers.docx [file pone.0337080.s001.docx]

**Response to reviewers**

**Background:**

This paper was first submitted to PLOS ONE in December 2024, with reference PONE-D-24-57831. However, there was a typographical error with the date that data collection for the study started, therefore the editorial office felt that our submission did not meet the ethical requirements of PLOS ONE’s human subjects research submissions.

**Reviewer’s comment:**

The Editorial Office provided the following reason for rejecting the manuscript:

*“We recognize that you obtained ethical approval from your local ethics committee after you began your study: your ethics approval document is dated August 28, 2020, while you began recruiting participants  in January 2020. You also state that informed consent was obtained from all participants, which indicates that the data were prospectively collected for research purposes. For human subjects research, PLOS ONE requires that authors obtain ethics committee approval before beginning the study, and we do not accept retrospective ethical approval.”*

**Response:**

I’m very sorry for this oversight. However, I would like to assure PLOS ONE that ethics approval for the study was granted in August 2020 and data collection started in January 2021. This correction has been made in the manuscript. I would also like to emphasise that there is no way we could conduct primary research in the University of Edinburgh without obtaining all the appropriate ethical clearance. I can confirm that ethical approval was granted before the commencement of this study.

Thank you for your understanding in this matter.
