# Supplementary material for: Evaluation of the delivery of the family nurse partnership programme in Scotland during the COVID-19 pandemic
Source: PLoS One. 2025 Dec 2;20(12):e0337080. doi: 10.1371/journal.pone.0337080 (PMC12671793; doi:10.1371/journal.pone.0337080)
Supplement: Attachment — Submitted filename: review plosone alimin alwi.docx [file pone.0337080.s002.docx]

**Abstract**

- An explanation of the methods used, results, and conclusions needs to be added

- Clarify the methods used in this study

- The research findings need to be emphasized

**Introduction**

- A detailed explanation of the research gap and the novelty of the research

- Clarify the objectives of the research

**Methods**

- Clarify the design of the methods

- Provide a detailed explanation of the data analysis used in this study

**Research Findings and Discussion**

- A more in-depth analysis of the data findings

- Present specific percentages and also present them in narrative form

- It is permissible to include more in-depth visual interpretations

- Explain the implications of the theory

**Conclusion**

- Add a few sentences explaining the research findings
