# Supplementary material for: Evaluation of the delivery of the family nurse partnership programme in Scotland during the COVID-19 pandemic
Source: PLoS One. 2025 Dec 2;20(12):e0337080. doi: 10.1371/journal.pone.0337080 (PMC12671793; doi:10.1371/journal.pone.0337080)
Supplement: Attachment — Submitted filename: Response to Reviewers..docx [file pone.0337080.s003.docx]

Professor Muhammad Abdul Rehman Rashid
Academic Editor
PLOS ONE

22^nd^ August 2025

**Manuscript ID:** PONE-D-25-02060

Dear Professor Muhammad Abdul Rehman Rashid,

**Re: Resubmission of manuscript entitled " Evaluation of the Delivery of the Family Nurse Partnership Programme in Scotland during the COVID-19 Pandemic”**

We would like to thank you and the reviewers for the time taken to review our paper. Please see our response to the reviewers’ comments below.

We would also like to seek exemption from making the data underpinning the findings publicly available for ethical reasons. Research ethics requirement does not permit the sharing of the research data we obtained from NHS patients and staff publicly. We would like to include the statement below as part of the publication.

“Ethical restriction by the Public Benefit and Privacy Panel for Health and Social Care (HSC-PBPP) in Scotland prohibits the authors from making the minimal data set publicly available because data contain potentially identifiable information which may compromise patient and staff privacy. However, request to access the data from the researchers could be made through the Ethics Committee via [phs.PBPP@phs.scot](mailto:phs.PBPP@phs.scot)”

This is also our updated funding statement: “The evaluation was funded by a grant from the Scottish Government, with reference number: C15968. The views expressed in this publication are those of the authors and not necessarily those of the funders. The funders had no role in study design, data collection and analysis, decision to publish, or preparation of the manuscript.”

We look forward to receiving a favourable outcome on our paper from you soon.

Yours sincerely,

Dr Lawrence Doi

**Response to reviewers**

| **Comment** | **Response** |
| --- | --- |
| **Reviewer #1** |  |
| 1. The study "Evaluation of the Delivery of the Family Nurse Partnership Program in Scotland during the COVID-19 Pandemic" provides valuable insights for the role of telehealth in nursing. Study is well structured and using quantitative and qualitative data make it more fruitful for community. Practical future recommendations are also worthy. | Thank you for this comment. |
| 2. Proof reading is required for grammatical errors and data should be available for more clarity. | We have now done a thorough proofreading of the manuscript. Unfortunately, NHS ethics requirement does not permit the sharing of our research data publicly, but request could be made through the ethics committee (phs.PBPP@phs.scot). We have sought exemption from the Editor. |
| **Reviewer #2:** |  |
| 1. All things considered, this paper significantly advances the assessment of the Family Nurse Partnership (FNP)'s implementation in Scotland during the COVID-19 pandemic, especially with regard to the telehealth service adaptation. To raise the manuscript's level of scientific and methodological quality, a number of important elements still need to be reinforced. | Thank you for this comment. |
| 2. The authors must include a thorough description of the study methodologies, including the approach used, the data collection strategies employed, and the sort of analysis done, in the abstract section. Furthermore, the study's results and first conclusions have not been made clear. It is important to highlight key findings so that readers are aware of the study's primary accomplishments right once. | We enhanced the description of the study methods and made the findings and conclusions clearer in the abstract. |
| 3. The author has given a thorough historical and geographical overview of the FNP implementation in the introduction. The research gap that underlies the necessity of this study, as well as the novelty or innovation this study offers in comparison to earlier studies conducted in other nations, such the UK, Canada, and the Netherlands, require a more thorough explanation. To make the research objectives more targeted and focused on the research problems that need to be addressed, they must also be defined, both generally and operationally. | We have provided further explanation of the research gap that our study addressed in relation to previous studies. We have also clearly articulated the aims of the study in both the abstract and at the end of the introduction section. |
| 4. Although the author refers to a mixed-techniques approach in the methods section, no specific explanation of the design—such as convergent, explanatory sequential, or embedded design—has been provided. The reasoning for this method's application should be described, as well as the steps taken to integrate qualitative and quantitative data. | We have clarified in the methods (and also in the abstract) that we used mixed-methods parallel design and explained the rationale for using this design. We have also explained the approach we used to integrate qualitative and quantitative data. |
| 5. Furthermore, a more thorough description of the data analysis methods should be included for both qualitative and quantitative data. This includes the software utilized, any validity or reliability standards, and the interpretation of the results. To help the reader comprehend the findings, the author has categorized them into major themes in the results and discussion part. However, the explanation of the results is typically descriptive and lacks a thorough analysis. The significance of the discovered data must be explained in greater depth, along with how it relates to the pertinent theory and context. | We have provided further description of the data analysis methods. We have also been explicit about the NVivo software and MS excel package we used to facilitate the data analysis. We have clarified how the survey questionnaire was developed and been explicit that no validated instrument was used.  We have now revised the findings and demonstrated how our findings link to pertinent theories and context where possible. |
| 6. In order for readers to fully understand the dynamics of the findings, the percentage of quantitative data that is supplied through surveys still uses broad language; instead, it needs to be extended in more narrative and specific terms. In order to enhance the argument and create a connection between the facts and the analytical narrative, data visualization tools like tables and graphs should be used in conjunction with more in-depth interpretations rather than just as visual aids. | We have now revised the findings and made the link between the interview and survey data more findings more explicit. We have included two tables and four figures to aid data visualisation, and in-depth interpretations added where possible. |
| 7. The author should also explain how these findings affect the development or reinforcement of the theory that underpins the FNP program, especially the theory of therapeutic partnerships in community health care. | We have discussed both in the findings and discussion sections how our findings relate to therapeutic relationship and other theories pertinent to the FNP programme. |
| 8. A few more phrases are required to clearly clarify the primary findings of this study, even though the author has indicated in the conclusion that telehealth has the potential to be employed in a hybrid fashion in the future when FNP is implemented. In order to provide more substantial value to the creation of community-based nursing care policies, the conclusion should include cover scientific contributions, real-world applications, and future research prospects. | The abstract and conclusion have been revised to clearly outline the scientific contributions, real-world applications and future research prospect. |
| 9. To be published in a respectable journal, this manuscript still needs to be improved in terms of goal formulation, data analysis depth, and methodological clarity, but overall it has the potential to be a significant contribution to the conversation on modifying maternal and child health services during the pandemic. | We hope the amendments we have made to the various sections address this point. |
| **Reviewer #3:** |  |
| Overall assessment   1. This study addresses an important question: how the Family Nurse Partnership (FNP) in Scotland adjusted to COVID‑19 restrictions and what that meant for service delivery. Although the topic is unquestionably relevant to public‑health practice, the manuscript reads more like an internal service report than a research article that meets PLOS ONE standards. The results are mostly descriptive, several methodological details are missing, and the paper does not yet comply with the journal’s data‑sharing policy. Significant revision is required before the work can be considered for publication. | Thank you for your overall assessment of our paper. We hope the amendments we have made to the paper based on your specific comments below and the other comments from the other reviews have addressed this point. |
| Major comments   2. Sample size and representativeness. Only fifteen client or family members were interviewed against thirty‑one nurses, and the manuscript gives no information on the number of eligible families, the proportion approached, or the reasons for non‑participation. In addition, the study covers eleven Scottish Health Boards, but the authors do not state how many Boards exist in total. Without these denominators it is impossible to judge representativeness or assess selection bias. | We have now included the number of eligible clients at the time of data collection under ‘sample’ in the methods. As we have indicated in the limitations, clients were recruited through their family nurses and ethics requirements prevented the research team from having direct contacts with clients, therefore it is difficult to say the reasons for non-participation. We have also clarified under ‘design, setting and context’ that we recruited from all the 11 health boards (out of a total of 14) currently running the FNP programme in Scotland. |
| 3. Research questions. The introduction reviews international literature but concludes with vague aims such as “evaluate” and “understand challenges”. Explicit research questions would give the study sharper focus. | Thank you. We have now clearly articulated the three main aims of the study. |
| 4. Quantitative component. The survey results are presented descriptively, without confidence intervals, hypothesis tests or any justification for the sample size. If inferential statistics are inappropriate, the authors need to explain why the survey was undertaken and clarify that its role is purely exploratory. | The survey was purely exploratory to support the qualitative results, and no inferential statistics were undertaken. |
| 5. Integration of methods. Qualitative and quantitative findings appear in parallel blocks and seldom interact. The paper would benefit from explicitly linking survey patterns. | We have revised the findings considerably and have explicitly linked the qualitative and survey findings. |
| 6. Rigour of the thematic analysis. There is no mention of an external audit of the coding framework, no assessment of inter‑coder reliability and no discussion of how emergent codes were incorporated. Greater transparency is needed to demonstrate analytical rigour. | We have provided further clarity of the analytical process and also added a paragraph on positionality as requested by reviewer number 4 |
| 7. Numerical detail. Key programme metrics, such as the reduction in home visits or the proportion of contacts delivered remotely, are discussed qualitatively but never quantified. The abstract in particular contains no numbers, making it difficult for readers to gauge the magnitude of change. | We have revised the findings considerably and where relevant we have provided figures to support the qualitative findings. We have also included a key survey result in the abstract. |
| 8. Statements on telehealth. The conclusion that telehealth “could play some role” is vague. The authors should offer concrete, operational recommendations—for example, criteria for hybrid scheduling or digital‑access assessments. | Thank you for this helpful comments. We have now revised and strengthened the conclusion. |
| 9. Data availability. The data are available only on request because of potential identifiability. This arrangement conflicts with PLOS ONE’s open‑data policy. At minimum, anonymised survey aggregates and redacted transcripts should be deposited in a public repository, or the authors must seek an exemption from the editors. | Unfortunately, NHS ethics requirement does not permit the sharing of our research data publicly, but request could be made through the ethics committee (phs.PBPP@phs.scot). We have sought exemption from the Editor. |
| 10. Timeliness. Data collection finished in 2021, yet the manuscript is being submitted four years later. The authors should explain why the findings remain current and describe any subsequent changes in FNP practice that might influence interpretation. | Unfortunately due to staff leaving posts the paper could not submitted earlier than we had hoped. However, we believe the findings are relevant to the delivery of the FNP programme post COVID-19. We have also included the following statement in the discussion section to describe current FNP practice in Scotland.  “As home visiting is still the predominant delivery mode in the 11 mainland Health Boards in Scotland post-COVID-19, telehealth may present an opportunity to expand the FNP programme to Island Health Boards and more remote and rural areas in Scotland not yet delivering the programme and other similar areas globally. |
| Minor comments   11. Demographic description. Governance rules may forbid disclosing individual‑level data, but an aggregated table of age bands, parity and deprivation quintiles is essential for readers to assess transferability. | We appreciate that demographic information would have enhanced the manuscript further. However, as we have noted in the limitations, information governance requirements prevented demographics data and deprivation data from being captured from the 15 clients that took part in the study. |
| 12. Selection bias. Clients were recruited via their own nurse, a process likely to introduce desirability bias. The limitation deserves a fuller discussion. | Addressed. We have now discussed desirability bias and recruitment in under the limitations. |
| 13. Typographical error. At line 63 the phrase reads “programme’s primary objectives of the programme”. The duplication should be removed. | Apologies for the oversight. Change now made. |
| **Reviewer #4:** |  |
| 1. The results are detailed and robust; however, including a brief summary of the main themes under each sub-section would enhance clarity. This would help readers follow the flow of the discussion more easily and better understand the similarities and differences between the findings from the qualitative interviews and the survey. | Thank you. We have revised the findings considerably and we believe this comment has now been addressed. |
| 2. Under the methods section, ensure that the missing components of COREQ (Consolidated Criteria for Reporting Qualitative Research) are provided. E.g. positionality. | Addressed. We’ve added an additional paragraph about positionality in the methods. |
| 3. Some grammatical errors are noticeable in the text. Kindly edit as appropriate. Few examples are on lines 28, 80, 101, 112, 114, 166. | Thank you. These have now been addressed. |
| **Additional reviewers’ comments from attachment** |  |
| **Abstract**  - An explanation of the methods used, results, and conclusions needs to be added  - Clarify the methods used in this study  - The research findings need to be emphasized | Thank you. We have revised the abstract considerably and we believe this comment has now been addressed. |
| **Introduction**  - A detailed explanation of the research gap and the novelty of the research  - Clarify the objectives of the research | We have provided explanation of the research gap and clearly articulated the research aims under the introduction. |
| **Methods**  - Clarify the design of the methods  - Provide a detailed explanation of the data analysis used in this study | Thank you. We have revised the methods considerably and we believe this comment has now been addressed. |
| **Research Findings and Discussion**  - A more in-depth analysis of the data findings  - Present specific percentages and also present them in narrative form  - It is permissible to include more in-depth visual interpretations  - Explain the implications of the theory | Thank you. We have revised the findings and discussion sections considerably and we believe these points have now been addressed. |
| **Conclusion**  - Add a few sentences explaining the research findings | We have revised the conclusions and we believe this comment has now been addressed. |
