# Supplementary material for: Evaluation of the delivery of the family nurse partnership programme in Scotland during the COVID-19 pandemic
Source: PLoS One. 2025 Dec 2;20(12):e0337080. doi: 10.1371/journal.pone.0337080 (PMC12671793; doi:10.1371/journal.pone.0337080)
Supplement: Attachment — Submitted filename: saran reviewer plos one.docx [file pone.0337080.s004.docx]

**Abstract**

The abstract already explains the background, methods, results, and conclusions, but it would be stronger if it added key figures briefly to emphasize the magnitude of the findings.

**Introduction**

The introduction has outlined the context of the FNP and reviewed previous research, but it would have been better if the emphasis on the research gap had been made more acute.

**Methods**

The methods section already explains the design, data collection techniques, and analysis, but it would be stronger if the explanation of the justification for the choice of method was expanded.

**Research Findings and Discussion**

The results section already presents qualitative and quantitative data with tables and visualizations, but it would be stronger if the interpretive analysis were expanded.

**Conclusion**

The conclusion already confirms the potential of telehealth in the FNP hybrid model, but it would be stronger if it added explicit statements regarding scientific contributions and practical implications.
