# Supplementary material for: Evaluation of the delivery of the family nurse partnership programme in Scotland during the COVID-19 pandemic
Source: PLoS One. 2025 Dec 2;20(12):e0337080. doi: 10.1371/journal.pone.0337080 (PMC12671793; doi:10.1371/journal.pone.0337080)
Supplement: Attachment — Submitted filename: Response_to_reviewers_auresp_2.docx [file pone.0337080.s005.docx]

Professor Muhammad Abdul Rehman Rashid
Academic Editor
PLOS ONE

29^th^ October 2025

Dear Professor Muhammad Abdul Rehman Rashid,

**Manuscript ID:** PONE-D-25-02060R1

**Manuscript title: " Evaluation of the Delivery of the Family Nurse Partnership Programme in Scotland during the COVID-19 Pandemic”**

We would like to thank you and the reviewers for the time taken to review our paper. Please see our response to the reviewers’ comments below.

We would also like to seek exemption from making the data underpinning the findings publicly available for ethical reasons. Research ethics requirement does not permit the sharing of the research data we obtained from NHS patients and staff publicly. We would like to include the statement below as part of the publication.

“Ethical restriction by the Public Benefit and Privacy Panel for Health and Social Care (HSC-PBPP) in Scotland prohibits the authors from making the minimal data set publicly available because data contain potentially identifiable information which may compromise patient and staff privacy. However, request to access the data from the researchers could be made through the Ethics Committee via [phs.PBPP@phs.scot](mailto:phs.PBPP@phs.scot)”.

This is also our updated funding statement: “The evaluation was funded by a grant from the Scottish Government, with reference number: C15968. The views expressed in this publication are those of the authors and not necessarily those of the funders. The funders had no role in study design, data collection and analysis, decision to publish, or preparation of the manuscript.”

We look forward to receiving a favourable outcome on our paper from you soon.

Yours sincerely,

Dr Lawrence Doi

**Response to Reviewers**

| **Reviewer #1** | **Response** |
| --- | --- |
| 1. Overall Assessment This is a relevant and timely study on the delivery of FNP during COVID-19. The design of mixed methods adds strength, but the document still needs improvements in clarity, methodological transparency and analytical depth to comply with PLOS One standards. | Thank you. |
| 2. Major Comments Methods: Provide more details about client recruitment, representativeness and how qualitative/quantitative data were integrated. | The recruitment and data collection under the methods, provide adequate details of how family nurses and clients were recruited. We have also added that the 90 nurses who took part in the survey represent a response rate of 41% across Scotland. We have also further explained the mixed-methods parallel (convergent) designed we employed, including how data were integrated. |
| 3. Analysis: Add clarity on thematic coding and justify why only descriptive survey data is used. Include confidence intervals if possible. | We have clarified the thematic analysis process we used.  The survey was purely exploratory, to support the qualitative findings and provide a broad snapshot to address the aims of the study, which were 1) to understand the features of telehealth employed to deliver the FNP programme during COVID-19 in Scotland; 2) to examine how FNP nurses and clients responded to the delivery of FNP through telehealth; 3) to evaluate the challenges of delivering the FNP through telehealth during COVID-19 and its implications for future delivery of the programme.  We have added the exploratory nature of the quantitative component as a limitation within the manuscript. |
| 4. Discussion: Go beyond the description: link findings to the theory, highlight the novelty compared to other countries and offer concrete recommendations for hybrid delivery. | In the previous round of revisions, we significantly revamped the discussion, linking it to theories underpinning the FNP. Point number 3 from reviewer #2 and point number 1 from reviewer #3 duly acknowledged this. However, we have further strengthened the discussion section to address the reviewer’s point. |
| 5. Data Availability: The current statement conflicts with the open data policy of PLOS One; Consider depositing anonymized aggregates or drafted transcripts. | Unfortunately, NHS ethics requirement does not permit the sharing of our research data publicly. Reviewer #2 also highlights that this explanation is plausible. We have also sought exemption from the Editor. |
| 6. Minor Comments Summary: Add sample sizes and a key numerical result; sharpen the conclusion with contribution/relevance of the policy. | The abstract clearly has sample sizes, and we have refined the abstract based on the previous reviewers’ comments. We have added a recommendation for policy. |
| 7. Introduction: Shorten background; emphasize the research gap. | Thanks for your comments. The introduction was revised based on comments from other reviewers and we strongly believe that shortening this at this stage will not fully address comments raised by other reviewers. The introduction clearly emphasizes the research gap that this study set out to address. |
| 8. Language: Necessary the final review; Avoid vague terms such as "many" or "several." | The use of terms such as “many” and “several” is commonly used to present qualitative findings as the use of numbers is generally not considered a good practice in qualitative e research because it **misrepresents the study's goal** of in-depth understanding, **implies misleading generalizability** from small samples, **strips context**, and creates a **false impression of precision** for subjective experiences. |
| 9. Conclusion: Provide clearer political implications and describe scientific contributions | Throughout the discussion and conclusion sections, we have highlighted the scientific contributions of the study as well as policy implications. |
| 10. Recommendation Strong potential, but needs clearer methods, deeper analysis and better alignment with the PLOS One requirements. | Thank you. We believe the revisions we have made address the second part of this point. |
| **Reviewer #2** |  |
| 1. The revised manuscript has significantly improved and now meets publication requirements. By clarifying the study design, strengthening methodological details, and increasing the depth of data analysis, the authors have addressed the concerns of previous reviewers. | Thank you. |
| 2. The inclusion of tables and figures, as well as a clearer connection between qualitative and quantitative results, has improved readability and interpretation. | Thank you. |
| 3. The study results are linked to relevant theory and practical implications for future family care provision. | Thank you. |
| 4. Although data are still limited due to ethical requirements, the explanations provided are plausible. | Thank you. |
| 5. Overall, the paper is well-written, technically sound, and provides important information on telehealth adaptations. | Thank you. |
| **Reviewer #3:** |  |
| 1. I appreciate the extensive revisions made to the manuscript. The authors have clearly addressed most of the concerns raised in the first review, and the paper is now much stronger in terms of methodological transparency, integration of findings, and clarity of conclusions. The inclusion of explicit research questions, numerical detail, and discussion of limitations all represent significant improvements. | Thank you. |
| 2. While I understand the governance restrictions, it would still be valuable for readers to see at least an aggregated table of client characteristics (e.g., age bands, parity, deprivation quintiles). Such information is essential for assessing the transferability of findings. If this is not possible, the limitation should be underscored more strongly in the manuscript. | Thanks for your comment. Unfortunately, we didn’t collect demographic data of the 15 clients who took part in interviews to protect participants confidentiality because of the small sample involved. Also, the goal was to focus on the experiences of clients receiving the FNP programme. We have acknowledged this as a limitation of the study in the manuscript. |
| **Reviewer #4:** |  |
| 1. Most of the comments have been addressed, though some are still not met. Such as: Justify replacing missing Gini data with the country average. Is this the standard practice or what is the rationale? | Thank you. We are pleased we addressed most of your comments.  We didn’t present any Gini data so we are unsure what this comment is referring to. |
| 2. DISCUSSION  In some paragraphs, authors need to contextualize the findings and draw adequate implications of the results. Also ensure to provide new interpretation of the study findings and not a repetition of the literature review in the previous sections of the manuscript. | In the previous round of revisions, we significantly revamped the discussion, linking it to theories underpinning the FNP. Point number 3 from reviewer #2 and point number 1 from reviewer #3 duly acknowledged this. However, we have further strengthened the discussion section to address the reviewer’s point. |
| **Additional reviewer’s comment from attachment** |  |
| **Abstract**  The abstract already explains the background, methods, results, and conclusions, but it would be stronger if it added key figures briefly to emphasize the magnitude of the findings. | Thanks for your comment. This comment was also raised in the previous round of revisions, and we revamped the abstract to include key figure from our findings. As this is a mixed methods study, we believe the abstract reflects our key findings. |
| **Introduction**  The introduction has outlined the context of the FNP and reviewed previous research, but it would have been better if the emphasis on the research gap had been made more acute. | Thanks for your comments. The introduction was revised based on comments from other reviewers in the previous round of revisions, providing a clear research gap that our study was designed to address. Please see rationale and aims of the study under the introduction. |
| **Methods**  The methods section already explains the design, data collection techniques, and analysis, but it would be stronger if the explanation of the justification for the choice of method was expanded. | We have provided rationale for the choice of the methods we used where relevant. |
| **Research Findings and Discussion**  The results section already presents qualitative and quantitative data with tables and visualizations, but it would be stronger if the interpretive analysis were expanded. | Thank you for your comment. In the previous round of revisions, we significantly revamped the findings and discussion. Point number 3 from reviewer #2 and point number 1 from reviewer #3 duly acknowledged this. However, we have further strengthened the discussion section to address the reviewer’s point. |
| **Conclusion**  The conclusion already confirms the potential of telehealth in the FNP hybrid model, but it would be stronger if it added explicit statements regarding scientific contributions and practical implications. | Throughout the discussion and conclusion sections, we have highlighted the scientific contributions of the study as well as policy implications. |
